# Volatile Constituents in Essential Oil from Leaves of *Withania adpressa* Coss. Ex Exhibit Potent Antioxidant and Antimicrobial Properties against Clinically-Relevant Pathogens

**DOI:** 10.3390/molecules28062839

**Published:** 2023-03-21

**Authors:** Mohammed Bourhia, Abdullah A. Alyousef, Ghizlane Doumane, Hamza Saghrouchni, John P. Giesy, Lahcen Ouahmane, Fatiha EL Gueddari, Yazeed A. Al-Sheikh, Mourad A. M. Aboul-Soud

**Affiliations:** 1 Department of Chemistry and Biochemistry, Faculty of Medicine and Pharmacy, Ibn Zohr University, Laayoune 70000, Morocco; 2 Department of Clinical Laboratory Sciences, College of Applied Medical Sciences, King Saud University, P.O. Box 10219, Riyadh 11433, Saudi Arabia; 3 Laboratory of Advanced Materials and Process Engineering, Faculty of Sciences, Ibn Tofail University, BP 242, Kenitra 14000, Morocco; 4 Department of Biotechnology, Institute of Natural and Applied Sciences, Çukurova University, 1380 Adana, Turkey; 5 Toxicology Centre, University of Saskatchewan, 44 Campus Drive, Saskatoon, SK S7N 5B3, Canada; 6 Department of Veterinary Biomedical Sciences, University of Saskatchewan, Saskatoon, SK S7N 5B4, Canada; 7 Department of Integrative Biology, Center for Integrative Toxicology, Michigan State University, East Lansing, MI 48824, USA; 8 Department of Environmental Science, Baylor University, One Bear Place #97266, Waco, TX 76798-7266, USA; 9 Laboratory of Microbial Biotechnology, Agro-Sciences and Environment (BioMAgE), Cadi Ayyad University, Marrakesh 40000, Morocco; 10 Laboratory of Chemistry, Biochemistry, Nutrition, and Environment, Faculty of Medicine and Pharmacy, University Hassan II, Casablanca 20000, Morocco

**Keywords:** natural products, phytochemicals, medicinal plants, antibiotic resistance, antimicrobial, free radicals

## Abstract

*Withania adpressa* Coss. ex is a plant used in traditional medications. Antioxidant, antibacterial, and antifungal properties of the essential oil from leaves of *Withania adpressa* Coss ex. (EOW) were investigated. EOW was extracted using a Clevenger apparatus, and its volatile compounds were characterized by GC-MS. Antioxidant potency was determined using DPPH, FRAP, and TAC assays. Antibacterial effects were determined vs. *Escherichia coli*, *Klebsiella pneumonia*, *Staphylococcus aureus*, and *Streptococcus pneumonia*; while its antifungal efficacy was determined vs. *Candida albicans*, *Aspergillus flavus*, *Aspergillus niger*, and *Fusarium oxysporum* using the disc diffusion and minimum inhibitory concentration bioassays. A chromatographic analysis showed that EOW contained eight phytochemical compounds constituting 99.14% of the total mass of oil. Caryophyllene (24.74%), Longifolene (21.37%), δ-Cadinene (19.08%), and Carene (14.86%) were predominant compounds in EOW. The concentrations required to inhibit 50% of free radical (IC50) values of antioxidant activities of EOW were 0.031 ± 0.006 mg/mL (DPPH), 0.011 ± 0.003 mg/mL (FRAP), and 846.25 ± 1.07 mg AAE/g (TAC). Inhibition zone diameters of EOW vs. bacteria were 18.11 ± 0.5 mm (*E. coli*), 17.10 ± 0.42 mm (*S. aureus*), 12.13 ± 0.31 mm (*K. pneumoniae*), and 11.09 ± 0.47 mm (*S. pneumoniae*), while MIC values were 51 ± 3, 47 ± 5, 46 ± 3 and 31 ± 1 µg/mL, respectively. Inhibition zone diameters of EOW vs. fungi were 31.32 ± 1.32, 29.00 ± 1.5, 27.63 ± 2.10, and 24.51 ± s1.07 mm for *A. flavus*, *C. albicans*, *F. oxysporum*, and *A. niger*, respectively. MIC values were 8.41 ± 0.40, 28.04 ± 0.26, 9.05 ± 0.76, and 22.26 ± 0.55 µg/mL, respectively. Importantly, the highest dose of EOW (1 mg/mL) showed negligible (~5%) cytotoxicity against MCF-12, a normal human epithelial cell line derived from the mammary gland, thus underscoring its wide safety and selectivity against tested microbes. To sum it up, EOW has exhibited promising antioxidant and antimicrobial properties, which suggests potential to abrogate antibiotic resistance.

## 1. Introduction

Natural products derived from herbs constitute an important source of therapeutic agents that have applications in folk pharmacopoeia [1]. The use of alternative medicine to treat illnesses is still encouraged by scientists and health organizations, despite the fact that modern pharmaceuticals have replaced several natural preparations historically used to manage diseases. [2]. Since some of the secondary plant metabolites used in modern medicine have been discovered through ethnobotanical investigations, empirical studies of various traditional plant preparations are useful for screening and selecting plants with medicinal activities [3]. For many years, medicinal and fragrant plants have been an important part of drug research and development. Chemicals produced by plants are used as therapeutics and raw materials for drug manufacturing and as models to design synthetic molecules used pharmacologically [4].

Because of the formation of free radicals during photosynthesis, antioxidant agents, including pigments like carotenoids, have been found in nearly all plants [5]. Free radicals play a major part in the development of tissue damage in people as they age or suffer from illnesses such as cancer, malaria, neurological disorders, and arteriosclerosis [6]. Antioxidants can prevent or mitigate certain diseases. Increasing attention has been paid to plant-derived antioxidant compounds because of their potential role in nutrition and health and sickness [7]. A wide variety of phytochemicals, such as phenolic or nitrogen-containing compounds, as well as carotenoids, are included in the category of naturally occurring antioxidants [8].

Human pathogenic microorganisms are a major source of disease and mortality worldwide. Even though in recent years the pharmaceutical industry has developed several new antibiotics, resistance to these synthetic antibiotics has increased to such a degree that now it is a health major issue globally [9]. The worldwide emergence of multidrug resistant (MDR) microbes is reducing the efficacies of currently available antibiotics and leading to major failures to treat diseases caused by these microbes [10]. Resistance of bacteria to chemically unrelated drugs is a growing health concern and can occur by over-expression of MDR efflux pumps [11].

Essential oils (EOs) are complex mixtures of molecules that have a low molecular weight and are extracted from plants by the process of steam distillation. Terpenoids and phenylpropanoids are the primary ingredients of EOs that give them their distinctive scent and biological activities [12]. Traditional systems of medicine across the globe use EOs to treat a wide range of health issues due to numerous medicinal properties attributed to these essential oils, including antibacterial, antioxidant, antifungal, anti-inflammatory, antimutagenic, antidiabetic, antiviral, antiprotozoal, spasmolytic, and anesthetic remedies [12,13]. While these properties have not changed much in the last several decades, new information has been gained on a few, most notably those related to antimicrobial modes of action [14].

The plant genus *Withania* has a long history of medicinal use, including the treatment of conditions, such as conjunctivitis, inflammation, stress, bronchitis, anxiety, neurological illnesses, ulcers, and liver disease [15]. The genus *Withania* possesses multiple pharmacological roles including anticholinesterase, analgesics, cures, immunomodulators, and antioxidants [16,17,18,19,20,21,22,23]. *Withania adpressa* Coss. ex (family Solanaceae), an herb commonly known by its English name of Winter Cherry, which grows in North Africa and in particular the Mediterranean basin [24], is known to exhibit pharmacological properties, including, antitumor, immunomodulatory, anticonvulsant, anti-inflammatory as well as anti-stress properties [25]. Few pharmacological properties of *Withania adpressa* Coss. ex have been investigated, and, therefore, the current study was undertaken to investigate the antioxidant, antibacterial, and antifungal properties of the essential oil from leaves of *Withania adpressa* Coss. ex.

## 2. Results

### 2.1. Phytochemical Identification of EOW

**Figure 1:**
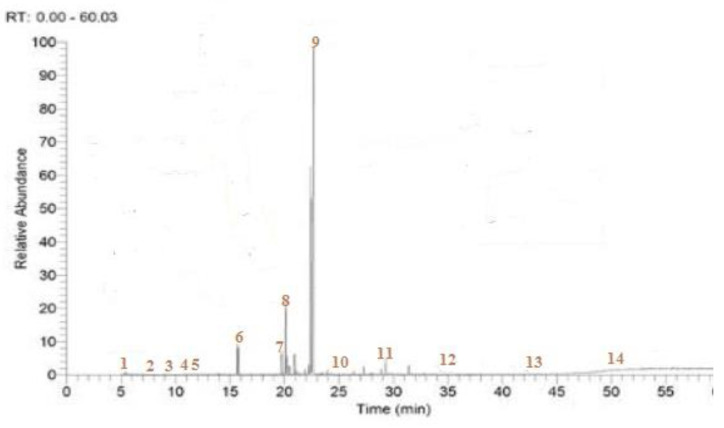
Chromatographic profile of EOW analyzed by GC/MS.

The yield of EOW obtained by hydro-distillation was 1.18% of the total mass of leaves extracted and comprised eight compounds, including Caryophyllene (20.26%), δ-Cadinene (18.08%), Longifolene (11.29%), and Carene (8.29%), which were the most dominant (Figure 1, Table 1).

### 2.2. Antioxidant Activity

**Figure 2:**
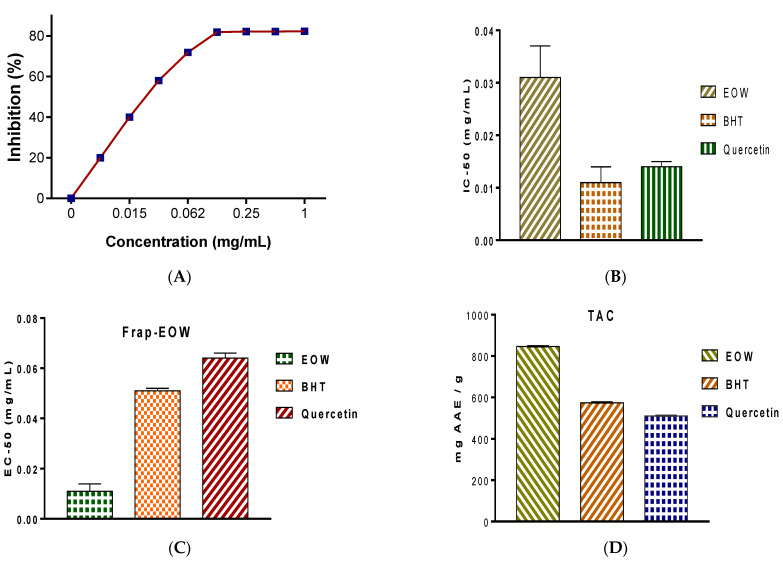
Antioxidant activity of EOW with positive controls: (**A**) Inhibition percentage of DPPH free radicals using various concentrations of EOW; (**B**) IC50 values of antioxidant activity of EOW using DPPH assay; (**C**) EC_50_ values of EOW obtained using FRAP assay; (**D**) Total antioxidant capacity of EOW given in mg of ascorbic acid equivalent per gram of EOW.

In a dose-dependent way, EOW was able to reduce the free radicals. The IC-50, which was calculated using the DPPH radical scavenging potency, was 0.031 ± 0.006 mg EOW/mL (Figure 2). The IC-50 was similar to that of EOs extracted from leaves of *W. frutescens* L., but more significant than what was shown with positive controls, such as BHT, which exhibited an IC50 of 0.011 ± 0.009 mg/mL, and Quercetin with an IC50 of 0.014 ± 0.001 mg/mL. The results of the FRAP experiment further indicated that EOW had substantial antioxidant efficacy with an EC_50_ value of 0.011 ± 0.003 mg EOW/mL, which was significantly less (more potent) than that of the positive control BHT, which had an EC_50_ of 0.051 ± 0.001 mg BHT/mL, and Quercetin for which the EC_50_ was 0.064 ± 0.002 mg/mL. Similarly, the results of the TAC demonstrated significant antioxidant potency of EOW, which was 846.25 ± 1.07 mg AAE/g EOW.

### 2.3. Antibacterial Activity

**Figure 3:**
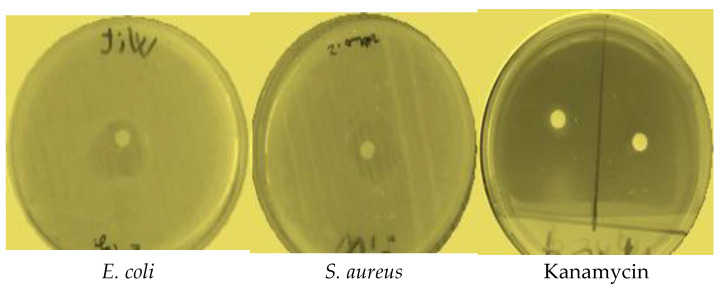
Photographs showing zones of inhibition of EOW with Kanamycin against bacteria.

EOW exhibited significant antibacterial activity against four species of bacteria: namely, *K. pneumonia*, *E. coli*, *S. aureus,* and *S. pneumoniae* (Table 2). EOW resulted in relatively large zones of inhibition against *E. coli* and *S. aureus* with diameters of 18.11 and 17.10 mm, respectively (Figure 3). More moderate antibacterial activities against *K. pneumoniae* and *S. pneumonia* were observed. Antibacterial activity was significant (*p* < 0.05) compared with that of commercial antibiotics Kanamycin and Oxacillin used as positive controls, neither of which was effective (Table 2). MIC values for EOW against *E. coli*, *K. pneumoniae,* and *S. pneumoniae*, ranged from 31 to 51 µg EOW/mL, which was excellent compared with MICs of Kanamycin and Oxacillin, which were 14 ± 1 and 15 ± 1 µg/mL, respectively.

### 2.4. Antifungal Activity

**Figure 4:**
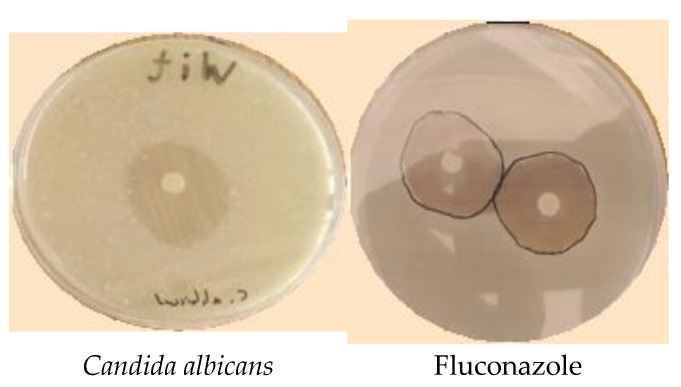
Photographs showing zones of inhibition of EOW along with Fluconazole against fungi.

EOW exhibited significant antifungal activity with large zone diameters against all fungal species, which reached 31.32 mm (Table 3) against three mold strains and one strain of yeast (Figure 4). The potency of EOW as an antifungal agent was similar to that of the commercially available fungicide Fluconazole which exhibited a strong antifungal activity with an exclusion diameter exceeding 33 mm for all strains of fungi tested. Similarly, MICs for EOW against *A. niger* or *C. albicans* were 22.26 and 28.04 µg/mL, respectively, which was less important when compared with those recorded for *A. flavus* (8.41 ± 0.40) and *F. oxysporum* (9.05 ± 0.76). These values were significantly (*p* < 0.05) greater (less potent) than the commercial fungicide Fluconazole, which had MICs values against all strains of fungi tested (Table 3).

### 2.5. In Vitro Toxicity of EOW against Human Cells

In order to investigate the safety and spectrum of WEO, the highest dose of 1 mg/mL was tested against MCF-12, normal human epithelial cells derived from the mammary gland. (Methods section). The highest dose of EOW (1 mg/mL) showed negligible (~5%) cytotoxicity against MCF-12 after 24 h incubation period (Supplementary Figure S1). This highlights its safety and selectivity against tested bacterial and fungal microbes.

## 3. Discussion

The yield of EOW of 1.18% on a mass basis, observed in this study for leaves of *W. adpressa* is reasonable when compared with that documented for another species in this genus, such as *Withania frutescens,* which had a yield of 0.28% [26]. This yield can be interesting when compared with some plants that are used industrially as sources of EOs, including *Garden Thyme* (2–2.75%), *bitter orange*, (0.5–1%), *Latin rosa* (0.1–0.35%), *English lavender* (0.80–2.8%), *sweet cumin* (1–3%), *Salvia rosmarinus* L. (1–2.5%), *Mentha balsamea* (0.5–1%), *Menth suaveolens* (0.79%) and *Menth arvensis* (0.36–1.36%).

The chemical composition of EOW was different from those of EOs from other plants in the genus *Withania*, particularly *W. frutescens* L., which had greater proportions of Carvacrol (31.870%), Thymol (30.076%), and Camphor (9.130%) [26]. However, several chemicals observed in EOW during this study were also observed in EOs of *Withania coagulans*, such as Caryophyllene (15%), Longifolene (12%), δ-Cadinene (11.7%), and Carene (11.3%) [27]. Relative proportions of chemicals in EOs are susceptible to variations due to edaphic and environmental factors [8].

The results of the DPPH, FRAP, and TAC bioassays demonstrating that EOW exhibited powerful antioxidant activity have been previously reported [28]. EOs of *W. frutescens* L. also exhibited significant antioxidant activities [28]. Results of recent studies have shown that the antioxidant capacities of herbal oils are sometimes greater than commercially available, synthetic antioxidants, including BHT and Quercetin [29]. Based on the chemical composition of EOW, the observed antioxidant potency may be attributed to the presence of the most common chemicals, all of which have been shown previously to have antioxidant potency. A review of pertinent literature revealed that Caryophyllene displayed strong antioxidant effects in both the DPPH and FRAP scavenging assays, with IC50 values of 1.25 ± 0.06 and 3.23 ± 0.07 µM for the DPPH and FRAP assays, respectively [30]. These findings are in line with the results of a previous study on the antioxidant efficacy of β-Caryophyllene [31]. In another study, Longifolene showed important scavenging activity vs. DPPH free radicals [32].

The fact that EOW showed substantial antibacterial action even at low doses vs. almost all strains is consistent with previously reported results [33] wherein it was stated that species of *Withania*, particularly EOs of *W. frutescens*, exhibited stronger antibacterial activity against *S. aureus*, *S. pneumoniae*, *E. coli,* and *K. pneumoniae*. Antimicrobial activities observed can be due to compounds detected in EOW by GC-MS, such as β-Caryophyllene, which is a sesquiterpene; Longifolene, which is a sesquiterpene hydrocarbon; δ-Cadinene, which is a sesquiterpene hydrocarbon; and 3-Carene, which is a monoterpene. These molecules are in the class of sesquiterpenes known to have antibacterial properties, particularly β-Caryophyllene, which has been reported to be active against causative agents of infections [30]. β-Caryophyllene has been shown to induce apoptosis through nuclear fragmentation and condensation pathways, including disturbance of mitochondrial membrane potential in cells [30]. Longifolene is a known antimicrobial compound [34]. δ-Cadinenes are a group of isomeric hydrocarbons, found in a wide variety of essential oil–producing plants. It has been reported that δ-Cadinene has a MIC value of 500 µg/mL vs. *S. pneumonia* [35]. 3-Carene is an antimicrobial compound that occurs in many plants and possesses ambiguous antibacterial mechanisms versus various pathogens. Antibacterial effects and mechanism of action of 3-Carene vs. the Gram-positive *B. thermosphacta* and Gram-negative *P. fluorescens* have been reported previously [36]. Microscopy analysis and the release of alkaline phosphatase revealed that 3-Carene causes damage to the morphology and wall structures of germs, which disrupts cellular processes and ultimately results in the death of bacteria [37].

The results of this study are consistent with those reported by other studies, which showed that constituents of EOs exhibit more activity collectively rather than individually, perhaps due to some synergistic mechanisms [38]. The fact that EOW showed promising antifungal activity against fungi like *A. niger, C. albicans, F. oxysporum, and A. flavus* is generally in line with what has been reported before, where EOs from *W. adpressa* weakly stopped *Penicillium italicum* from growing. [39]. Similarly, EOs of *W. frutescens* were found to be active vs. *A. niger*, *C. albicans, F. oxysporum,* and *A. flavus* [26]. Modes of antifungal action of EOs are multifaceted and dependent on absolute and relative proportions of compounds present [8]. Multiple studies show that phytochemicals included in essential oils may disrupt cell membranes and affect a wide range of cellular functions. Reduced membrane potentials, proton pump interruption, and exhaustion of ATP could all play a role in the antifungal activity observed [40]. It is possible for antifungal drugs to exert their effects by causing damage to the cell membrane rather than by impairing the metabolic processes of the fungus, which would ultimately result in the death of the fungus [41]. Fungicidal effects of EOs can be linked to monoterpenes, which have been speculated to act as solvents of cell membranes owing to their lipophilicity, by directly disintegrating the lipidic component of the plasma membrane of the microbe. Subsequently, this leads to an increased extracellular conductivity and leakage of cell constituents [41,42]. Owing to their natural origin, EOs have been reported to exhibit wider safety margins and negligible toxicity, relative to synthetic agents and drugs. Therefore, they have been classified by the U.S. Food and Drugs Administration (USFDA) as GRAS (Generally Recognized as Safe) [43,44].

## 4. Materials and Methods

### 4.1. Chemicals

Muller Hinton (MH) agar, Muller Hinton Broth (MHB), Peptone Glucose (YPG) agar, Peptone Glucose broth, Potato Dextrose Agar (PDA), and Potato Dextrose Broth (PDB) were used as culture media. For the disk tests, positive control inhibitors included Kanamycin (KAN), Oxacillin (OXA), and Fluconazole (FLU). All media are supplied by Biokar, Pantin, France.

### 4.2. Plant Material

*Withania adpressa* Coss. ex was collected from the Moroccan Sahara (29.7509° N, 7.9756° W) in March 2021, at the peak of flowering. After being authenticated by a botanist, the plant was placed at the University (USMBA-Morocco) Herbarium under the reference A2/WDBF21. Next, leaves were washed with water and dried in a dark, ventilated space for seven days before extraction.

### 4.3. Extraction of Essential Oils

Leaves were crushed using a blender to yield 200 g of powder. Next, the powder was soaked in 700 mL water prior to extraction of essential oil using a Clevenger for 2 h under boiling. Afterward, the obtained EOW was stored in the dark at 5 °C until further use. The dry weight of the plant matter was used to figure out the EOW yield in percentage [29,45].

### 4.4. Characterization of Phytochemicals

Identification and separation of volatile compounds in EOW were conducted using GC-MS. The analysis was carried out using a gas chromatograph (Agilent GC 6850) equipped with an injector connected to a mass spectrometer (Agilent 5973 with ion trap) with an electronic impact ionization mode (70 eV) with a scanning interval of one wave and a fragmentation order of 5 × 10 to 4.5 × 10^3^ daltons. Separation of individual compounds was performed using a 30 m, DB-5 capillary column of 0.2500 mm internal diameter with a film thickness of 0.250 mm. Temperature-programmed gas chromatography was initially 40 °C for 2 min followed by 260 °C for 10 min, then increased by 20 °C/min to 280 °C, and finally held at 280 °C for 10 min. The injection temperature was 200 °C and the temperature of the interface was 300 °C. With a flow rate of 1.4 mL/min, helium served as a transport gas. For chemical analysis, hexane (1% *v*/*v*)) was used to dilute EOW (1 µL). The solvent delay was less than 4 min, and the total duration of the chromatographic (GC/MS) analysis of EOW was about 59.81 min. EOW volatile chemicals were identified by a comparison of their retention indices to those reported in the literature (namely, the Adams database) and confirmed by masses of their parent ions and the fragment grams. The retention indices for the *n*-alkanes used (C9–C40) were obtained by GC/MS under the same analytical circumstances as for the EOW. The formula for calculating the retention index was as follows [46]:

$$KI=100n+100\left[\left(Trx-Tn\right)/Trx+1-Tn)\right]$$

Tn represents the retention time of the *n*-alkane that comes before the target compound, and *Trx* + 1 represents the retention time of the *n* + 1 alkane that comes after the target peak. Each peak’s quantitative component was given a percentage [47].

### 4.5. Antioxidant Activity

In vitro antioxidant potency of EOW was conducted using three chemical assays: DPPH, FRAP, and TAC.

#### 4.5.1. DPPH Free Radical Scavenging

The DPPH test was carried out by mixing 100 µL of various concentrations of EOW, varying from 0.001 to 1 mg/mL, with 0.75 mL of freshly prepared 0.4% DPPH solution. After 30 min of dark incubation, the absorbance was measured at 517.00 nm. BHT and quercetin were used as positive controls, while methanol was used as a negative control. The efficacy of EOW was evaluated by determining the concentration required to inhibit 50% of DPPH free radicals (IC_50_) [29,48]. The percentages of DPPH radical inhibition were calculated (Equation (1)).

Inhibition (%) = [(Ac − As)/Ac] × 100 (1)

where Ac—Absorbance of the negative control; AS—Absorbance of the test sample.

#### 4.5.2. Ferric Reducing Power

Two-point fifty milliliters of potassium ferricyanide and phosphate buffer (1%) were mixed with EOW (1 mg/mL) [49]. The mixture was then heated to 50 °C for 20 min. Afterward, 2.50 mL of trichloroacetic acid was put in the reaction medium before centrifugation.

Finally, each concentration was thoroughly mixed with 2.50 mL of distilled water and 0.5% FeCl3 before measuring the absorbance at 700 nm. In this experiment, a blank reagent with no sample was employed as a negative control, while BHT and quercetin were utilized as the reference standard. The efficacy was assessed by determining the concentration required to achieve a 50% antioxidant effect (EC-50) using a calibration curve.

#### 4.5.3. Total Antioxidant Capacity of EOW

A volume of 0.1 mL EOW was poured into one milliliter of 1.2 N sulfuric acid, 28 mM Na_3_PO_4_, and 4.0 mM (NH_4_)2MoS_4_ [50]. After that, the solution was placed in a 95 °C water bath and incubated for 90 min before reading the absorbance at a wavelength of 695.00 nm. We utilized a reagent blank as a negative control, while positive controls included BHT and quercetin. The overall antioxidant efficacy of EOW is given as mg EAA/g.

### 4.6. Antibacterial and Antifungal Activities

#### 4.6.1. Microbial Strains

The antibacterial potency of EOW was tested on a total of eight different microorganisms, including four different strains of bacteria—namely, *Escherichia coli, Klebsiella pneumonia pneumonia, Staphylococcus aureus,* and *Streptococcus pneumoniae*; while *Candida albicans, Aspergillus flavus, Aspergillus niger,* and *Fusarium oxysporum* were selected for the antifungal test.

#### 4.6.2. Microbial Suspension Preparation

For the inoculation of bacterial and yeast suspensions, 2 to 3 colonies from a fresh culture of MH and YPG media were taken and suspended in 0.9% NaCl solution.

Thereafter, the optical density of the obtained suspensions was verified using a UV-visible spectrophotometer at wavelength λ = 625 nm. Consequently, the bacterial suspension was determined to be approximately 1-2 × 10^8^ CFU/mL, while the fungal suspension was 1-5 × 10^6^ CFU/mL [51]. 

Inocula for molds were prepared as described elsewhere [52]. Briefly, sporulation was obtained by culturing the fungal strains on a PDA medium at 28 °C for 7 days. Spores were collected by flooding the dishes with 5 mL of 0.05% (*v*/*v*) Tween 20, using a sterile spreader. Spores in the suspension were counted using a hemocytometer (area: 0.0025 mm^2^; depth: 0.2 mm) with light microscopy before the suspension was adjusted to 10^6^ conidia/mL in 0.9% NaCl solution.

Sporulation was achieved by growing the fungal strains on the PDA medium for seven days at a temperature of 28 °C. To collect the spores, a sterile spreader was used to cover the surface of the plates with 5 mL of Tween 20 diluted to a volumetric concentration of 0.05%. Before adjusting the concentration to e 10^6^ conidia/mL in 0.9% normal saline, the spores in the suspension were counted using a hemocytometer (area: 0.0025 mm^2^; depth: 0.2 mm) with light microscopy.

#### 4.6.3. Disc Diffusion Method

Sensitivities of microbial strains to EOW were determined using the disc diffusion method as described previously [53]. First, 1 mL of fresh microbial cultures was added to each Petri dish containing either an MHA, a YPG, or a PDA medium in a 90 mm Petri dish, and then the plates were allowed to stand for 10 min. After that, sterile discs with a diameter of 6 mm were impregnated with 5 microliters of EOW, or positive controls, such as Kanamycin (50 μg), Oxacillin (4 μg), and Fluconazole (500 mg), were deposed on surfaces of media in Petri dishes. Finally, inoculated Petri dishes were then kept in the dark at 30 °C for the fungal species and 37 °C for the bacterial species. For bacteria and fungi, the inhibitory rates were calculated 24 and 48 h after incubation in mm [18].

#### 4.6.4. Determination of Minimum Inhibitory Concentration (MIC)

To determine MICs, the micro-dilution method was conducted as previously described [54]. First, EOW was diluted in 5% DMSO, while a positive control, such as Kanamycin, Oxacillin, and Fluconazole, was diluted in YPG or MHB media; then 50 μL of each medium was poured into microplate wells. Following that, one hundred microliters from each fraction was poured into the first well. After that, a micro-dilution was performed by diluting the sample by a factor of 2 in each well, with the exception of the last well, which acted as the positive control for growth. Inoculation was accomplished by placing fifty microliters of the suspension into each well of the microplate, with the exception of the first well, which acted as a control for the absence of growth. The bacterial strains were kept in microplates for 24 h at 37 °C, while the yeast strain was kept in an incubator at 30 °C. After incubation, 20 μL of 1% 2, 3, 5-triphenyl tetrazolium chloride (TTC) (Biokar, France) was added to microplate wells. After incubation, 20 µL TTC (1%) was added to each well of the microplate. After two hours of incubation, the wells in which bacteria grew became pink due to the activity of dehydrogenases, but the wells in which bacteria did not grow remained colorless The MIC was determined to be the lowest concentration at which there was no pink hue.

The antifungal activity of mold strains, expressed as MICs, was evaluated using the macro-dilution bioassay [52]. After diluting EOW in a PDB medium, tubes were inoculated with 100 μL of fresh fungal conidia previously adjusted to 10^6^ conidia/mL. The tubes were incubated under agitation for 5 days at 27 °C. After incubation, the MIC was defined as the least concentration at which no growth of fungi was visible. Following the dilution of EOW in the PDB medium, the tubes were infected with 100 µL of fresh fungal conidia that had been adjusted to a concentration of 106 conidia/mL beforehand. At a temperature of 27 °C, the test tubes were kept in the incubator for five days. Following incubation, the MICs were determined to be the lowest concentrations at which there was no detectable fungal growth.

### 4.7. Cell Line, Culturing Condition and MTT Viability Assay

MCF-12 (ATCC^®^ CRL-10782™), a cell line derived from the human mammary gland (epithelial breast), was obtained from the American Type Culture Collection (ATCC, Manassas, VA, USA). Dulbecco’s Modified Eagle Medium (DMEM)/high glucose supplemented with two mM _L_-glutamine, 10% FBS, and 1% penicillin/streptomycin was utilized to grow cells. Next, 80–90% confluent cultures were trypsinized and split to an optimal seeding ratio. Cells were cultured at 37 °C in 5% CO_2_ in a humidified incubator. The highest dose of WEO of 1 mg/mL was prepared in DMSO, and its cytotoxicity against the MCF-12 cell line was conducted according to our previously published protocol using 3-[4,5-dimethylthiazol-2yl]-2.5-diphenylterazolium bromide (MTT) assay [26].

### 4.8. Statistical Analyses

Means and standard deviations of triplicate tests were used to represent the results of this study. Tests for normality and homogeneity of variance were carried out using Shapiro–Wilk and T-tests, respectively. Multiple comparisons were handled utilizing the ANOVA and Tukey’s HSD test as a post hoc analysis of the variance test. At *p* less than 0.05, the difference was considered significant.

## 5. Conclusions

Results of the studies reported here on chemical composition, antioxidant, antibacterial, and antifungal properties of EOs from leaves of the Winer Cherry, *Withania adpressa* L., demonstrate their promising antioxidant and antimicrobial potential, which could render these EOs useful as natural medicinal products to deal with resistance to other antibiotics; however, potential toxicities of the EOs to humans and nontarget organisms need to be investigated prior to their pharmacological uses. Importantly, the highest dose of EOW (1 mg/mL) has shown negligible (~5%) cytotoxicity against MCF-12, normal human epithelial cells derived from the mammary gland, thus underscoring its wide safety and selectivity against tested microbes. Our findings highlight the significance of employing EOW as a safe and selective alternative to synthetic antibiotics to override the frequently encountered antimicrobial resistance.

## Figures and Tables

**Table 1:** Identification of phytochemicals in EOW by GC/MS.

Peak	Retention Time	Compound	Retention Index		Chemical Formula	Cas	Area (%)
			Calculate	Literature			
1	5.40	Acetonyl acetone	924	921	C_6_H_10_O_6_	110-13-4	2.05
2	7.51	δ-2-Carene	1002	1002	C_10_H_16_	554-61-0	8.29
3	9.07	Santolina alcohol	1040	1040	C_10_H_18_O	35671-15-9	3.25
4	11.48	Undecane	1100	1100	C_11_H_24_	1120-21-4	4.28
5	11.56	Phenyl propanal	1102	1102	C_9_H_10_O	53-S3-8	2.61
6	15.65	Ethyloctanoate	1195	1197	C_10_H_20_O_2_	106-32-1	6.35
7	18.77	Nonanoic acid	1269	1270	C_9_H_18_O_2_	112-05-0	7.73
8	20.10	Acetophenone	1296	1298	C_9_H_10_O_2_	98-86-2	8.58
9	22.66	Caryophyllene (z)	1408	1415	C_15_H_26_O	7S737-41-2	20.26
10	24.91	Longifolene	1409	1407	C_15_H_24_	475-20-7	11.29
11	29.72	γ-Cadinene	1517	1522	C_15_H_24_	39029-41-9	18.08
12	35.99	Cadalene	1674	1675	C_15_H_18_	483-78-3	0.12
13	43.47	Rimuene	1894	1896	C_20_H_32_	1686-67-5	0.08
14	50.33	Methyl linoleate	2081	2085	C_19_H_34_O_2_	112-63-0	5.17
		Total identified					99.14

**Table 2:** Diameters of zones of inhibition and MICs for EOW, Kanamycin, and Oxacillin against strains of bacteria.

Strain	Diameter of the Inhibition Zone (mm)			Minimum Inhibitory Concentration (µg/mL)		
	EOW	Kanamycin	Oxacillin	EOW	Kanamycin	Oxacillin
*E. coli*	18.11 ± 0.5	0	0	51 ± 3 ^a^	15 ± 1 ^b^	11 ± 0 ^b^
*K. pneumoniae*	12.13 ± 0.31	0	0	46 ± 3 ^a^	14 ± 1 ^ns^	14 ± 1 ^ns^
*S. pneumonia* *e*	11.09 ± 0.47	0	0	31 ± 1 ^a^	15 ± 1 ^ns^	13 ± 1 ^ns^
*S. aureus*	17.10 ± 0.42	0	0	42 ± 5 ^a^	14 ± 1 ^ns^	14 ± 1 ^ns^

The values in rows with the same letters did not differ significantly (*n* = 3, one-way ANOVA; Tukey’s test, *p* = 0.05). ns, non-significant.

**Table 3:** Diameters of zones of inhibition and MICs for EOW against fungal strains.

Strain	Diameters of Inhibition Zones (mm)		Minimum Inhibitory Concentrations (µg/mL)	
	EOW	Fluconazole	EOW	Fluconazole
*A. niger*	24.51 ± s1.07 ^c^	36.12 ± 2.04 ^c^	22.26 ± 0.55 ^c^	3.21 ± 0.04 ^c^
*C. albicans*	29.00 ± 1.5 ^b^	33.08 ± 1.23 ^b^	28.04 ± 0.26 ^b^	2.44 ± 0.08 ^b^
*A. flavus*	31.32 ± 1.32 ^a^	39.41 ± 1.21 ^a^	8.41 ± 0.40 ^a^	2.52 ± 0.03 ^a^
*F. oxysporum*	27.63 ± 2.10 ^a^	32.52 ± 1.20 ^a^	9.05 ± 0.76 ^a^	3.68 ± 0.04 ^a^

The values in rows with the same letters did not differ significantly (*n* = 3, one-way ANOVA; Tukey’s test, *p* = 0.05).

## Data Availability

All data pertinent to this work are included in the paper.

## Supplementary Materials

The following supporting information can be downloaded at: https://www.mdpi.com/article/10.3390/molecules28062839/s1, Figure S1: Cytotoxicity of EOW against MCF-12, MCF-12, a normal human epithelial cells derived from mammary gland.
