# Supplementary material for: Volatile Constituents in Essential Oil from Leaves of Withania adpressa Coss. Ex Exhibit Potent Antioxidant and Antimicrobial Properties against Clinically-Relevant Pathogens
Source: Molecules. 2023 Mar 21;28(6):2839. doi: 10.3390/molecules28062839 (PMC10056193; doi:10.3390/molecules28062839)
Supplement: Supplementary file 1 [file molecules-28-02839-s001.zip › molecules-2196180-supplementary.pdf]

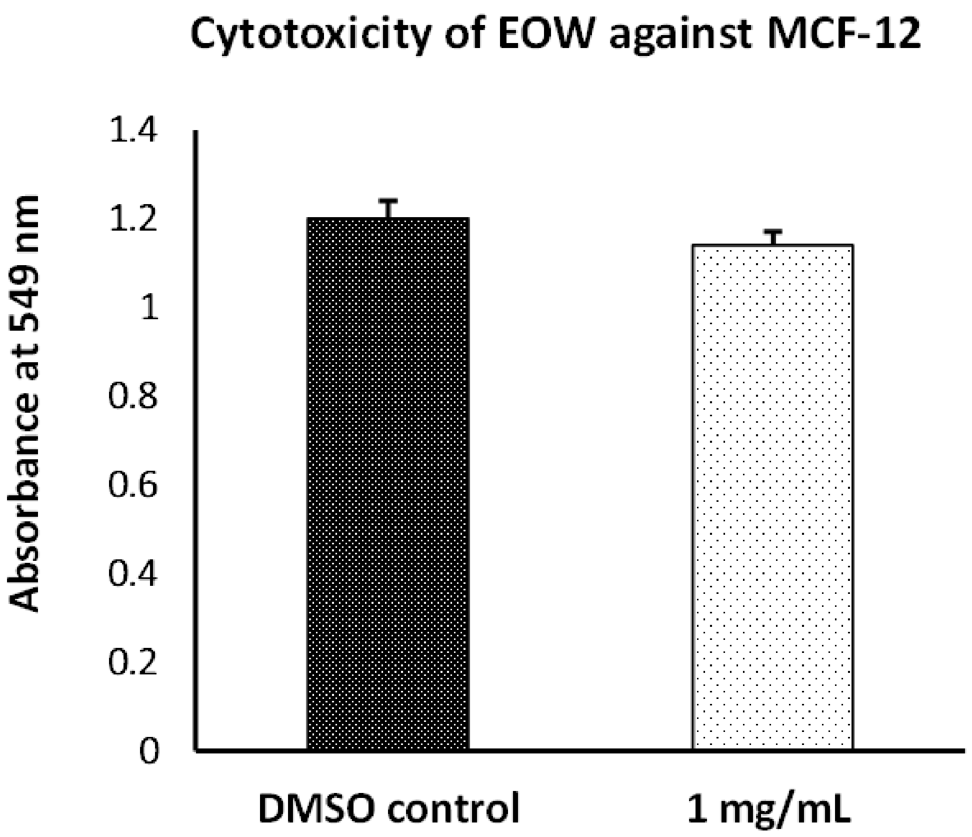

Supplementary Figure S1. Cytotoxicity of EOW against MCF-12, MCF-12, a normal human epithelial cells derived from mammary gland. Cells were treated with the indicated concentration of EOW (1 mg/mL) with DMSO control for 24 h followed by determination of viability by MTT assay, as detailed in the Methods section. Data represent mean ± SD of 8 technical well-replicates.
